# Corrective biology: psychosomatics in and as neuropsychoanalysis

**DOI:** 10.1136/medhum-2019-011645

**Published:** 2019-06-19

**Authors:** Felicity Callard, Constantina (Stan) Papoulias

**Affiliations:** 1 Department of Psychosocial Studies, Birkbeck University of London, London, UK; 2 Health Service & Population Research, Institute of Psychiatry, Psychology & Neuroscience, King's College London, London, UK

**Keywords:** psychoanalysis, affective neuroscience, Jaak Panksepp, interdisciplinarity, psychosomatic

## Abstract

This article analyses how and with what consequences body–mind relations (the sphere of the psychosomatic) are being modelled in the 21st century through considering the interdiscipline of neuropsychoanalysis. The promise of the term psychosomatic lies in its efforts to rework standard, bifurcated models of mind and body: somatic acts are simultaneously psychic acts. But neuropsychoanalysis, as it brings the neurosciences and psychoanalysis together to model an embodied ‘MindBrain’, ends up evacuating another potent characteristic found in much of the psychosomatic tradition—its refusal to adjudicate, a priori, what counts as the adaptive or well-regulated subject. The psychosomatic problem in psychoanalysis profoundly disturbs everyday models of functionality, adaptation and agency, by positing the psyche as an ‘other’ of the physiological within the physiological. By contrast, neuropsychoanalysis ends up parsing too easily the healthy from the pathological body, such that it is only the latter that is subject to forces that work against self-preservation and self-regulation. In so doing, neuropsychoanalysis recasts the radical problematic that the psychosomatic installed for psychoanalysis in the direction of a corrective biology. This corrective biology is given form in two ways: (1) through translating the Freudian drive—that unruly and foundational concept which addresses the difficult articulation of soma and psyche—into a series of Basic Emotion Systems modelled by the affective neuroscientist Jaak Panksepp and (2) through resituating and quarantining the troubling, non-adaptive aspects of the Freudian psyche within the domain of addiction. That easy separation between the healthy and the pathological is all too often found in current descriptions of healthcare and patient encounters. The article refuses it and calls for the revivification of other ways of thinking about how human subjects—psychosomatic organisms—find ways to live, and to die.

How is the mind to recognise itself in, say, an act of vomiting? The real mystery is not ‘the mysterious leap from mind to body’: how something mental, an idea, can cause something physical, like vomiting, to occur. The real mystery is how it is possible for there to have been no leap. How could vomiting itself be ‘thinking’? How could we recognise the mental in something so physical?1

Jonathan Lear, in his philosophical explication of psychoanalysis as the attempt to imagine what a science of subjectivity might be, uses the example of vomiting to revisit Freud’s famous description of the ‘puzzling leap from the mental to the physical’.2 That leap for Freud was best exemplified through his account of hysterical conversion, and Felix Deutsch later took up both the leap and a model of conversion in *On the Mysterious Leap from the Mind to the Body*.3 Lear challenges a model in which ‘the mental’, as a distinct sphere of action, comes to *cause* something physical, and instead sets our sights on the strangeness of vomiting as itself thinking. Such a claim demands an ontological and epistemological shift. The ejection of matter carries meaning; it raises questions of relationality and intention, of what is wished outside or inside. These are well in excess of a straightforward need to rid the body of physiologically registered toxins. What might vomiting be if we choose not to interpret it either as simply an involuntary muscular spasm or the effect of a mental act? In challenging *bien pensant* understandings of bodies and body–mind relations, the invitation to see vomiting as thinking suggests, instead, that there is an ‘other’ of the physiological within the physiological. How would one unravel the kind of thinking—ferocious, destabilising or melancholic—that occurs as and through vomiting? What kind of body, in other words, is the subject who vomits, or shakes or faints?

The desire to disrupt standard, bifurcated models of mind and body can be found in many strands of research and practice that are in some way indebted to psychoanalysis. This article focuses on the strand which calls itself neuropsychoanalysis. As its name testifies, neuropsychoanalysis is an attempt to map psychoanalysis through a certain kind of neuroscience, grounding the Freudian unconscious through neurobiological correlates, thereby opening up a location for the psyche–soma (the *psychosomatic*) which would revitalise both partners. We use the term psychosomatic throughout this article in a capacious way—as a difficult term with numerous histories and one which signals attempts to account for how the psyche and the body are intimately bound up with one another. We consider the neuropsychoanalytic project in light of the concern that this special issue (on biopolitics and psychosomatics) has with how the assumed divisions between mind and matter, and the mental and the somatic, have been both a provocation and a lure for analysis and for therapeutics. In this context, we are interested in understanding how neuropsychoanalysis arranges mind and matter, nature and value through an explicit commitment to install subjective intentionality *within* rather than alongside biology.4

While medical humanities as a field has so far engaged relatively little with neuropsychoanalytic texts, this work has received some analytical attention within the humanities more broadly.5 And some writers have shown interest in the emerging interdiscipline as a space to advance the medical humanities. For example, the philosopher Catherine Malabou, in *The New Wounded: From Neurosis to Brain Damage*, argues that neuropsychoanalysis ‘recognises the essential and reciprocal link between the life of the brain and subjective experience’.6 In particular, Malabou is interested in how neuropsychoanalysis ties psyche, affects, body and brain together—by placing the regime of sexuality and that of cerebrality *on the same plane*. Equally, the writer Siri Hustvedt discusses the influence of a neuropsychoanalytic group on her development of a psychosomatic account of the ‘mysteries of [her] own nervous system’ consequent on a shaking fit she experienced while giving a public talk about her father (who had died 2 years previously).7 We see, here, how neuropsychoanalysis is being put to work with reference to the question of the psychosomatic: it appears to promise a way of overcoming the sequestration of brain (matter) from mind, of affect from cognitive intentionality, of sexual logics from cerebral logics, of the corporeal from the mental.

We maintain that the neuropsychoanalytic commitment to an embodied subjective intentionality is actualised via recourse to a neuroevolutionary backdrop that plays a determining role in both the interpretation of experimental findings and the solidification of overarching concepts. While neuropsychoanalysis is currently a relatively minor enterprise (both within the neurosciences and within psychoanalysis), we argue that its substantial claims carry significance for how the relationship between psyche and soma is being envisaged in the 21st century. This is because, first, neurospsychoanalysis interprets the Freudian unconscious through the conceptual apparatus of a certain kind of affective neuroscience and thereby posits not only a new foundation for the psyche–soma but notably one that, its proponents argue, demonstrates fidelity to Freud’s own neurological origins. Second, neuropsychoanalysis recasts the problematic that the psychosomatic installed for psychoanalysis in the direction of what we are calling a corrective biology. While the psychosomatic problem in psychoanalysis profoundly disturbed everyday models of functionality, adaptation and agency—see also Monica Greco’s contribution to this special issue8—the ontologies of the subjective which are advanced by neuropsychoanalysis end up parsing too easily the distinction between the healthy and the pathological body. It is only the latter that is conceived as moving against self-preservation and self-regulation. Both these neuropsychoanalytic moves threaten to evacuate what is most potent in that powerful strand of psychoanalytic and psychosomatic writings which has refused to adjudicate, a priori, what counts as the adaptive or well-regulated subject.

In what follows, we discuss how neuropsychoanalysts make use of affective neuroscience—and the research of Jaak Panskepp in particular—to translate that most troublesome and antagonistic of psychoanalytic concepts, the Freudian drive, as a series of Basic Emotion Systems (BESs), which propel bodies to engage with and respond to their environment. This translation, which produces an ‘embodied brain’ as a new foundation for the psyche–soma, comprises, we argue, the most important joist that has allowed the neuropsychoanalytic structure to gain enough solidity and methodological consistency to propagate itself as a community and as a body of knowledge—even as many of its concepts and theories remain contested. We focus on the Freudian drive not only insofar as it names one of the most foundational concepts within psychoanalysis, but also because drive theory is Freud’s attempt to address the relationship between the psyche and the body by pulling the so-called ‘puzzling leap’ in the opposite direction. That is, the drive is a ‘concept on the frontier between the mental and the somatic’—it designates a turnpike at which the psyche emerges from and acts on the body.9 In the final part of the article, we briefly consider how neuropsychoanalysis presses its reworked drives—now hooked into Panksepp’s Basic Emotion Systems—into the complex question of addiction. This elaboration of addiction is an exemplary instance in which neuropsychoanalytic accounts install the embodied brain by cleaving apart appropriately and inappropriately (pathologically) activated subjects—subjects motivated by life-affirming pleasures from those whose drive has been hijacked by something other than self-preservation. We ask what it might mean to refuse that easy separation, one which is all too often found in current descriptions of healthcare and patient encounters, so as to revivify other means of thinking about how human subjects—psychosomatic organisms—find ways to live and to die.

## Neuropsychoanalysis and the embodied brain

Neuropsychoanalysis is a diverse, interdisciplinary field that comprises the efforts of researchers and clinicians within several branches of psychoanalysis, the neurosciences and psychiatry to construct a shared space of inquiry in which ‘to consider how empirical findings and neuroscientific theories can be enhanced by metapsychological knowledge derived from subjective, clinical observation and vice versa’.10 It was named and constituted by a small group of researchers at whose heart lies the psychoanalyst and neuropsychologist Mark Solms. Solms was one of the founding editors of the journal *Neuropsychoanalysis*, the launch of which in 1999 constituted the first formal use of the term neuropsychoanalysis to demarcate a field. While neuropsychoanalysis as a self-described field is thus a 21st century phenomenon, its emergence enacts a particular turn within a much lengthier set of engagements between psychoanalysis and the neurosciences.11 Solms himself, frequently in collaboration with Karen Kaplan-Solms (also a psychoanalyst and neuropsychologist), has been working since the 1980s to bring to light the extent of Freud’s own neuroscientific and neuroanatomical research, prior to his decisive turn away from neurology in the 1890s.12 The main neuropsychoanalytic claim is that consciousness must be understood as both affective and embodied and that the Freudian theory of the psychic apparatus, while falling short in bringing together mind, brain and body, nevertheless provides us with the foundations for such an understanding (notably the first congress of the fledgling discipline in 2000 was on the topic of emotion).13 For Solms, Freud’s model of the psychical apparatus, is grounded in, and through, affect—even as the affective ‘basement’ exists in Freud *despite* Freud’s own predilection for higher level, ‘cognitively oriented’ models.14 These important psychoanalytic insights were neglected, however, on Solms’ account, by the ‘behaviourist juggernaut’ and, then, subsequently, by the information-processing excesses of cognitivism.15 As neuropsychoanalysis emerged, then, Solms and his close collaborators consciously imagined it both as a return to Freud’s affective emphasis, and as a fulfilment of Freud’s own ambition for a bridge between the neuronal and the psychic. In producing such a bridge, neuropsychoanalysis then also comes as a response to the rallying call made by the Nobel Prize-winning neuroscientist Eric Kandel for the convergence of disciplines addressing the mind. In 1999, Kandel pointed to the ‘excitement and success’ surrounding current biology and proleptically described ‘a unified discipline of neurobiology, cognitive psychology and psychoanalysis’ that would be capable of ‘forg[ing] a new and deeper understanding of mind’.16 In the introductory issue of *Neuropsychoanalysis*, Solms and Nersessian announced that the journal’s goal was ‘to create an ongoing dialogue with the aim of *reconciling* psychoanalytic and neuroscientific perspectives on the mind [italics added]’17 and explicitly employed the concept of ‘consilience’18 and the language of a ‘unity of purpose’.19 It is the language of bridges, consilience, dialogue, unification and reconciliation that comes to demarcate the space of the inter-, or a movement across.

## Panksepp and affective neuroscience

It is on the terrain on which and through which the psychosomatic was itself elaborated—that of the affects and of their intimate entwinement with the somatic—that neuropsychoanalysis is built. At the first Neuropsychoanalytic Congress in 2000, Solms argued that:

[W]e have to find a point of contact between contemporary neuroscience and psychoanalysis, which will most likely be at the level of [a] basic theory of the basic mechanisms of emotion. If we can find a level where the language and concepts of contemporary neuroscience match the language of psychoanalysis, we will have a foothold… 20

For Solms and his collaborators, affective neuroscience provides such a foothold: it is a theory marking out the inter-implication of psyche and soma, an inter-implication which Freud had attempted to engage through his conceptualisation of the drives. The figure who enabled this translation was Jaak Panksepp, a leading affective neuroscientist whose research focused on affect in non-human animals.21 Solms and his collaborators need Panksepp because affective neuroscience is able to provide a theory of the ‘basement’. Solms is thereby able to find his foothold in Panksepp’s formulation of Basic Emotion Systems: ‘nomothetic endogenous behavioural and affective resources of the organism’.22 Nearly 20 years on from Solms’s call at the first neuropsychoanalytic congress, these ‘nomothetic endogenous’ resources of the organism are now hard baked into much of the neuropsychoanalytic architecture. Panksepp’s formulations will ensure that Solms is able to lay out a tidy basement, one fully aligned with self-preservation and self-regulation. This has forestalled the elaboration of what we argue would be more supple means through which neuropsychoanalysis might articulate how human subjects, as psychosomatic entities, are riven by complex affective relationships both with their own suffering and with the milieu in which they are embedded.

Panksepp offers a vision in which affects, far from being epiphenomenal, are central to what ‘motivate[s] the organism to promote its survival and reproductive success’.23 His argument, developed in his 1998 book *Affective Neuroscience*, emerged through his research on *unconditioned* behaviours in animals, that is, on behaviours which do not originate in priming and learning tasks.24 Panksepp posited that mammals engage in action in the world as a response to a series of endogenous and discrete ‘primal “feeling networks”’.25 He distinguished seven such feeling networks or Basic Emotion Systems (BESs), with specific yet interlocking sites in the subcortical regions of the brain. Neurochemical activity across these regions generates different adaptive patterns of behaviour: mating behaviour (LUST), fight or flight responses (FEAR and RAGE), separation anxiety (PANIC), social bonding (CARE) and rough and tumble (PLAY).26 Notably, one of these systems, which Panksepp called SEEKING, underlies and enables the others, constituting an originary orientation towards the world, an exploratory disposition. While nominally a feeling network, SEEKING does not have a specific feeling tone proper to it but acts as a booster of sorts to the other basic emotions. SEEKING is not reactive to specific external stimuli: rather, it is plugged into bodily need detectors and is the means by which homeostatic imbalances turn into an urge to call forth objects which would redress them.27 Panksepp calls this urge ‘a goad without a goal’.28 Its prototypical instantiation is mammalian foraging behaviour, exemplified in Panksepp’s experiments by the whisker-twitching rat sniffing out the space around her. SEEKING is a kind of global ‘anticipatory excitement’, an adaptive twitching which orients mammals to the world and predisposes them towards ‘life-supporting affordances of the world’ through a euphoric bribe.29

Solms and his various collaborators make use of Panksepp to rewrite the Freudian drives as the foundation of the ‘embodied brain’ (which they call ‘MindBrain’). This is indeed at the heart of their project to undo the mind–body bifurcation, to rework what the psychosomatic might mean. To this end, they make use of Freud’s oft-quoted definition of the drives from ‘Instincts and Their Vicissitudes’ as ‘the psychical representative of the stimuli originating from within the organism and reaching the mind, as a measure of the demand made on the mind for work in consequence to its connection with the body’.30 In the neuropsychoanalytic rewriting of the drive, the stimuli are the physiological imbalances in the visceral body, which are felt as qualitatively expressed pressures (basic emotions) orienting the subject towards a very specific kind of work. This work is making use of objects in the world that would relieve such pressures and still the body’s clamour. For neuropsychoanalysis, then, drives, mapped on to Panksepp’s BESs, serve to *regulate* homeostatic imbalances by motivating the organism to search for objects appropriate for the correction of these imbalances.31

## The trouble of the drive

Yet, this emphasis on the drive as a means of regulating the visceral body does not fully address the full purpose of Freud’s work on drive theory. The path of the drive (*Trieb*) in Freud is particularly complex. Freud famously described the theory of the drives in his ‘New Introductory Lectures on Psychoanalysis’ as ‘our mythology’: drives ‘are mythical entities, magnificent in their indefiniteness. In our work we cannot for a moment disregard them, yet we are never sure that we are seeing them clearly’.32 The reference to mythology concerns the later, more speculative and more frequently contested aspects of Freud’s work, which introduce the duality of life and death drives, or Eros and Thanatos. However, this leap to the mythological, while marking the moment in Freud’s work where he is arguably at the furthest from clinical observation, is in fact made in an attempt to negotiate a paradoxical persistence that is borne out within clinical observation, namely, that the psyche’s work can in no way be wholly captured by the vital register of self-preservation, nor indeed by the call to pleasure understood as satisfaction of need and consummation of tension. At the heart of Freud’s elaborations regarding the origin and vicissitudes of the drive is his interest in the origin and vicissitudes of fantasy. As Jean Laplanche and Jean-Betrand Pontalis made so clear in their 1968 essay on fantasy and psychic reality, it is only by detaching sexuality ‘from any natural object’ and ‘hand[ing] [sexuality] over to fantasy’ that sexuality might start existing in psychoanalysis *as* sexuality.33 That fantasy, and psychic reality, threaten perpetually to be lost or marginalised in psychoanalytic metapsychology across the decades provides evidence of how difficult it is to envisage the profound challenge fantasy offers to the standard bifurcation that is made between the categories of the real (the material) and of the imaginary. As we shall see, the manner in which neuropsychoanalysis attempts to articulate the psyche–soma means that fantasy is either elided or its strange potency vitiated.

In this regard, we should be clear that Freud’s mythological moment in relation to the drive was his third attempt to theorise his persistent conviction that the psyche’s work can in no way be wholly captured by the vital register of self-preservation, and, with it, to understand the relationship between the body’s vital needs and that attachment to a certain suffering. Freud initially conceived of drives as bodily demands related to sexual excitation by provisionally distinguishing, in 1905, the sexual drive from other biological needs (such as the need for nourishment).34 In 1910–1911, he then bifurcated the drives through opposing sexual and life preservation elements (now instantiated as ego-drives).35 Finally, in 1920, Freud offered the more ‘mythic’ opposition between the life and death drives, supplemented by their composites.36 In this last opposition, self-preservation was integrated with a certain sexuality under the auspices of Eros, both working to regulate bodily needs of different kinds. However, this integration was once again countered by a certain principle of dysregulation, now operating in the name of Thanatos, the death drive, a repetition compulsion that undoes the regulatory hypothesis of the pleasure principle. What persists across the different iterations of the drives is the notion that the psychic apparatus is animated by a conflict, even as the contours and stakes of this conflict change. In the first instantiation, drives diverge from bodily needs and sexuality from self-preservation. In the second, this divergence remains, but now self-preservation is also folded into the level of drive; while in the third, the mythology of Eros and Thanatos provides the metapsychological framework for a radical negativity introduced by the repetition compulsion, a negativity that is not simply at variance with self-preservation, but which specifically operates as its undoing. Indeed, one could venture that the psyche, rather than naming an ‘other’ of the soma, is the very instantiation of this conflict as such. What persists in Freud, and is articulated with increasing clarity in his later work, is a sense that there is something fundamental in and to the subject which undoes the subject, or, one might say, there is a persistence of and attachment to suffering that lies beyond the pleasure principle. And this is an unhomely attachment. Freud, in imagining the response to the ‘improbability of [his] speculations’, as he considers the ‘impulsion to self-destruction’, offers a startling image of the psychosomatic: ‘A queer instinct, indeed, directed to the destruction of its own organic home!’37 The queer and complex vision that Freud sketches here—in which the organic is host to a force that runs fundamentally counter to its hospitality—is, we shall see, one that will not be replicated in neuropsychoanalysis. There, we find instead what might be described as an orthotic logic—one driven by a commitment to a certain kind of normativity in terms of the regulatory dynamics of the human subject. That orthotic logic will attempt to straighten things out and put the soma and the psyche back in their place.

Furthermore, and contra Stratchey’s English-language translation of Freud’s *Trieb* as ‘instinct’, an extensive body of scholarship has shown that drives are not the same as biological instincts.38 Freud argued in his ‘Three Essays on the Theory of Sexuality’ that heterosexual intercourse is not biologically programmed, but an outcome of a certain ‘civilising’ of the drive, one which is never guaranteed or finalised. Indeed, not only do drives lack the pre-fitted objects typical of instincts but, crucially, this contingency of their object is framed by the dynamics of repetition: the finding of an object figures as a return to a lost object in which there is—unavoidably and painfully—a phantasmatic dimension. As the psychoanalyst André Green has argued: libido originates ‘[i]n the interlacing of impulse and phantasy’: ‘where the impulse brings energy, whether expended or pent up, the phantasy acts as a vector orienting and directing,…through the object and through narcissism’.39 This phantasmatic element is indispensable. That the drives are not instincts opens, too, a deep cleft between psychological and behavioural accounts, on the one hand, in which self-preservation drives the entirety of the organism, and psychoanalysis. As Freud indicated in his posthumously published expository and synthetic work ‘An Outline of Psycho-Analysis’:

The power of the id expresses the true purpose of the individual organism’s life. This consists in the satisfaction of its innate needs. No such purpose as that of keeping itself alive or of protecting itself from dangers by means of anxiety can be attributed to the id.40

This cleft between the psychological and the psychoanalytic is, we argue, closed by neuropsychoanalysis. A prudent management of the drive makes Freud’s drive much more congruent with standard scientific, adaptational accounts. Solms, for example, in a section on *Trieb* in his discussion of the forthcoming revised standard edition of Freud, appears to encourage such congruence by arguing that ‘The scientific meaning of “drive” is also constantly evolving’, and ‘[c]urrently … appears to imply narrower forms of homeostatic regulation of the bodily economy than Freud, perhaps, intended in his usage of *Trieb*’.41 What we witness in the neuropsychoanalytic recasting of Freud is that the register of self-preservation becomes, contra Freud, the task of the psychic apparatus in its entirety.42

While this neuropsychoanalytic translation expands the bodily dimension of Freud’s definition of the drive, and carries forward elements of all three iterations of his drive theory, it does so at a price. It curiously elides the oppositional dynamic, the conflict which animates all iterations, and the tenacity of which necessitates Freud’s iterations in the first place. Indeed, in the neuropsychoanalytic gloss, bodily needs are not split off from sexuality (the first iteration of Freud’s theory of the drives). On the one hand, sexual pleasure (Panksepp’s LUST) becomes one of many pleasurable interactions with the world;43 on the other, the foundational dimension of the drive, its pressure (*Drang*), now expressed as SEEKING, provides an underlying, undifferentiated orientation to the world in the service of all bodily needs, whether they be emotional or gastric (the prototypical instantiation of SEEKING for Solms and Panksepp is, after all, animal *foraging* behaviour, and they term the function of the system ‘appetitive’). Equally, the opposition between sexuality and what Freud called ego-drives or self-preservation (the second iteration of Freud’s theory of the drives) is weakened here. To the expanded bodily realm of positive affects and pluralized satiations, Solms and his collaborators juxtapose a sober guardian:

[the] embodied, instinctual brain—must of necessity be constrained by the cognitive brain and its predictive modeling… 44

The reference to the ‘cognitive brain’ signals a return to the body–mind split via a distinction between the pleasure and reality principles. But this is a curious and diminished return: the embodied brain, as mapped through Panksepp’s BESs, and through SEEKING in particular, delivers a rather odd pleasure principle, which is no longer equivalent to Freud’s own.45 This is because in Panksepp’s system there is an assumed primal yoking between pleasure and self-preservation: as Mark Solms and Margaret Zellner state, ‘to the extent that our vital needs are met, satisfied, we feel pleasure’ (indeed the BESs are biologically encoded, phylogenetic memories of this yoking).46 We would do well here to remember the extensive body of psychoanalytic and psychosomatic literature that demonstrates how the satisfaction of ‘vital needs’ so frequently does not bring the human subject the feeling of pleasure.

But that is not the tradition, of course, in which Panksepp’s research is embedded. For it is important to remember that SEEKING is defined as powering up exploration for ‘things that will ultimately propagate our genes’.47 In a sense, Panksepp and Solms’s evolutionary reading of the drives folds them within a narrative of self- and species preservation, and means that these constitute the reality principle *avant la lettre*. Indeed, this configuration is perhaps closest to Freud’s concept of a life drive—or to Eros, in his third and more speculative iteration of the drive theory, one which similarly mobilises the pleasure principle in the direction of self-preservation. However, again, Freud postulated Eros in his attempt to address the curious insistence in clinical observation of an attachment to suffering, or of forces beyond the pleasure principle. This insistence of the non-adaptive in the subject—one of the names for which is the death drive—itself marks the perpetual problem at work in the drive. That a certain kind of self-destruction lies at the heart of psychic functioning has also been of profound importance to psychosomatic investigations—for example, in those of the Paris School of Psychosomatics.48 But what happens if this insistence of the non-adaptive is refused? When, for example, the neuropsychoanalytic elaboration of the domain of the psychosomatic is underwritten by an affective neuroscience organised around the urges characterising mammalian foraging behaviour? In the final part of our article, we read neuropsychoanalytic models of addiction as one important attempt to address and—crucially—exorcise the refractory problem of the conflictual drive, and of the non-adaptive that lies at the heart of the human subject. If the neuropsychoanalytic elaboration of the embodied mind claims to dissolve the divide between matter and mind, it simultaneously, and relatedly, commits to a divide between the appropriately regulated, and distressingly dysregulated, subject.

## Malfunctioning bodies: addiction and unreal objects

The neuropsychoanalytic reorientation of Freudian drive theory as the expression of an expanded, correctly adaptive body means that any non-adaptive activation of the BESs and of the SEEKING system in particular, that is, an activation that neither ensures the survival of the organism nor contributes to reproductive success, needs to be understood through a reference to malfunction and not as something inherent to the embodied brain.

In a 2011 review paper with the compelling subtitle ‘Why depression feels so bad and what addicts really want’,49 some of the key proponents of neuropsychoanalysis discuss a series of such malfunctions in a reading of the (then) latest neurobiological findings on addiction. This they present as a definitive elaboration on the question of motivation and drive, as well as an updating of Freud. Margaret Zellner, Douglas Watt, Mark Solms and Jaak Panksepp argue that addictions constitute a pathological dysregulation of the organism’s emotional systems, both appetitive (SEEKING) and consummatory (LUST, CARE and PLAY), which they term a ‘hijacking’ of the drive. Crucially, here addictions are pathological insofar as they are—as neuropsychoanalysts put it—*pseudo*-appetitive. That is, addicts satisfy their urges in the absence of ‘real’ objects in the world. What is meant by ‘real’ here is very specific: it is a worldly, functional affordance which supports life by supporting regulation. The substances addicts use are not construed as ‘real’ because they feed (increase) craving but do not satisfy ‘biological needs’. For example, ‘opiate-induced hedonic fog’ generates a feeling of satisfaction as an ‘empty (objectless) pleasure.’50

Not incidentally, the authors relate the pleasure of addiction to that of compulsive masturbation to argue that they are both ‘pleasure without attachment or worse: substitutive pleasure in the absence of a specific longed-for object’.51 In the concluding statements of their paper, they turn to Freud’s early 1898 paper ‘Sexuality in the Aetiology of the Neuroses’,52 arguing that he regarded masturbation as a ‘“primal addiction” that may serve as a substitute for mature sexual relations’,53 such that the masturbator’s urges can only be addressed by the ‘reestablishment of “normal sexual life”’.54 In line with Freud’s suggestions, they propose that the addict too can be restored through a re-engagement with life-giving affordances provided by ‘social attachment’ as well as ‘all other rewarding aspects of loving interaction’.55 Thus, addictive and masturbatory pleasures alike represent a hijacking or pseudo-activation of urges/drives, insofar as they operate outside *a biologically fitted purpose*—that is, they do not promote social and reproductive fittedness, respectively. This equation of the real with the biologically fitted purpose qualifies and delimits Panksepp and Solms’ insistence on the primary objectlessness of the BESs and differentiates such objectlessness from Freud’s insistence that the object of the drive is contingent.56 For Panksepp and Solms, BESs do not have a fixed object but they do motivate and reward an orientation to the world and its life-giving affordances. For Freud, satisfaction, or indeed excitation, does not bear a necessary relation to self-preservation or species preservation: indeed, the uncoupling of the subject from self-preservation is *what the psyche does*. Psychoanalysis is a (potentially) interminable project precisely because this relation is not a given but has to be forged. Because of this, any object can come to be seized by the drive—including, of course, the subject’s own body. In this sense, the object of the drives is never quite ‘real’. It is insofar as the object is de-routed from its functional purpose and becomes fantasmatically invested that the object affords satisfaction (whether that object is a fetishist’s shoe, the unseeing eye of the hysteric described in Freud’s essay ‘Psychogenic Visual Disturbance According to Psycho-Analytical Conceptions’, or a child’s cotton reel gleefully recovered from its precipice only to be destroyed yet again). The yoking of the Freudian drives to biological fittedness in neuropsychoanalysis, and the positing of addiction and masturbation as a ‘derangement’ of the drives, effectively sets up psychic conflict as secondary. It therefore becomes avoidable through prudent (self-)management, rather than as that which, in psychoanalysis, constitutes the psyche in the first place.57

## Corrective biology

Neuropsychoanalysis is already being described as that which takes up the mantle of 20th-century psychosomatics. Joseph Dodds has argued that neuropsychoanalysis addresses ‘the philosophically ancient discussion of the relation of mind and body, an issue at the heart of psychoanalysis from the start with its early work on hysterical conversion… and later developments in the psychosomatics of the Paris School and beyond’.58 Meanwhile, as we complete this article in 2019, Mark Solms is on the point of publishing a new, revised, English language standard edition of Freud.59 Solms, who has had a long-held interest in translations of Freud’s work60 has, notably, indicated that in his role as translator he has ‘added some remarks on neuroscientific aspects of the Project for a Scientific Psychology’ and *The Interpretation of Dreams*’.61 It is therefore not far-fetched to imagine that the engine of neuropsychoanalysis will gain energy through the manufacture of a new English-language Freud for a new neuropsychoanalytic age. While it is impossible to know prior to its publication how far neuropsychoanalytic styles of thought might inflect this translation, it is evident from Solms’s already published writings that he has felt free to import neuropsychoanalytic terms indebted to affective neuroscience as equivalents for Freud’s own terms. Crucially, this importation includes the term ‘basic emotion’—a phrase signifying, as we have shown in our discussion of Panksepp, an inbuilt affect, and one radically at odds with psychoanalytic models of drive.62

Psychoanalysis, since the moment of its birth, has been defined by the question of translation. The drive is a kind of translation of the somatic to the psychic; Freudian repression has been theorised by the psychoanalyst Jean Laplanche as a failure of translation;63 psychoanalysis translates concepts and phenomena, in the process transforming and defamiliarising them, and psychoanalysis is itself perpetually translated into other idioms and other languages. Neuropsychoanalysis comprises one more in the series of translations of psychoanalysis, through bringing psychoanalytic writings and epistemologies into intimate contact with experimental research in the life sciences. It is always important to take heed of what is discarded, occluded or transformed in acts of translation. The neuropsychoanalytic translation of Freud has rightly insisted on the embodied nature of the subject. But it has, we argue, through its commitment to the BESs, ended up removing the ‘other’ of the physiological within the physiological. For it has ended up sequestering fantasy from biology—and thereby has removed one of the most provocative possibilities for imagining what the terrain of the psychosomatic might be.

Neuropsychoanalysis effects this removal at the very moment at which it pursues its aim to install subjective intentionality within the physiological. Mark Solms, in his foreword to a series of case studies by the psychosomatic clinician Jean Benjamin Stora, emphasises the importance of Stora’s contributions by noting that they have been central to ensuring the ‘integration into physical medicine of the mental apparatus’.64 Here the field of neuropsychoanalysis appears at first glance as the protector of the radical insights of psychoanalysis in its redrawing of the perimeter and topology of the mental such that the mental burrows deep within the soma. But Solms then adds that the mental apparatus is ‘a “system” not fundamentally different from the other functional systems of the body, such as the digestive and respiratory tracts’.65 Here, and across neuropsychoanalytic texts more broadly, we see a flattening whereby the mental apparatus is imagined as being of the same order as—and as epistemically available—as the functional systems of the body. Such a move, we argue, while appearing to lay the foundations for an engagement with the psychosomatic, introduces such an engagement bereft of the challenge that Freud’s invocation of the psychic apparatus, per se, installed. If the mental apparatus, like the other systems of the body, is returned to a familiar kind of functionality, this precisely disavows the otherness denoted by the Freudian unconscious, which is brought to life through hysterical conversion.

Neuropsychoanalysis is fond of arguing that it aims to complete the project that Freud himself ‘was forced—through lack of pertinent knowledge—to abandon’.66 Here, neuropsychoanalysis is visualised as a ‘cleaned up version’ of psychoanalysis—one that has ridded itself of Freud’s predilection for the speculative or the mythological, as well as his commitment to scientific paradigms that were outdated even at the moment in which he was writing. But in ridding psychoanalysis of the mythological, neuropsychoanalysis also ends up disavowing that which the mythological was called to account for: that the work of the psyche cannot be wholly subsumed within the work of self-preservation and that the pleasure of the psychosomatic organism is not secured by the satisfaction of vital needs.

The use of Panksepp’s affective neuroscientific foundations is able, on Solms and Zellner’s account, to replace Freud’s ‘unfortunate tendency’ to cast affective ‘inherited structures in Lamarckian terms’,67 with a more robust, evolutionary framework. The proponents of neuropsychoanalysis we have been discussing here claim that once one has stripped out these unfortunate tendencies from psychoanalysis, it is possible to discern that Panksepp and Freud are proposing *essentially the same thing*. Such a claim is possible because the full range of data and conceptual architecture that is drawn into the orbit of neuropsychoanalysis—the empirical, laboratory-based findings, the insights drawn from the clinic, the parts of Freud that are deemed of use—are interpreted through the lenses of self-preservation and reproductive fittedness.

But this meta-discursive lens relies on a neuroevolutionary ‘back story’ that is, we suggest, just as mythological as that of Freud’s own evolutionary drama (with its ‘unfortunate’ Lamarckian tendencies, its primal horde, and its primal father). The anthropologist Allan Young has argued in his own research on the neurosciences that ‘[m]yth is not antithetical to science’ and has employed the concept of the back story—‘a fictive or notional history that precedes the events described in the narrative’—to indicate how a ‘plausible genealogy of the social brain’ has been pieced together.68 The ‘back story’ of the embodied brain in neuropsychoanalysis is far less narratologically complex and without the dialectical turns that characterise the back story that Young has pieced together in relation to social neuroscience. The embodied brain’s back story presupposes that ‘the emotional brain systems that generate…affects have existed since the dawn of mammalian brain evolution’,69 such that ‘our common ancestor was already feeling powerful emotions long before humans walked the face of the earth’.70 On such an account, ‘[G]enes that create emotional brain networks can’, moreover, ‘figuratively be seen as the keepers of raw emotional memories—of retained dispositions and capacities that enable mammals to survive and to thrive’.71 Neuropsychoanalysis wishes to strip from Freud his vision of ‘primal me walking with a primal horde’ fearing a ‘primal daddy coming with the scissors to cut-off our primal willies’, so as to be left with Panksepp’s account of affect as ‘pre-programmed physiological and behavioural responses of great biological value signified by specific varieties of pleasurable or unpleasurable experience’.72 The mythological figure of the ‘primal me’ no longer has a ‘primal daddy’ wielding a primal knife; she is, rather, the ancestral rat from the dawn of mammalian evolution whose SEEKING system comprises her ‘future-*opportunity* orient(ation)’ and provides her with the vital *joie de vivre* to keep her foraging and topping up her food supplies.73 The normativity that underpins the commitment to reproductive fittedness functions as a meta-discourse that subtends and determines the neuropsychoanalytic readings of findings and concepts emerging from both the neurosciences and from psychoanalysis. The queer, psychosomatic vision of an organism that might experience urges to destroy ‘its own organic home’—which Freud sketched out in his lecture on ‘Anxiety and instinctual life’—is excised.

## Conclusion: neuropsychoanalysis and the psychosomatic

The feminist cultural theorist Elizabeth Wilson, in a series of powerful expositions on the domain of the psychosomatic, has demonstrated what might be possible if we break away from thinking and working with categorical models that quarantine the physiological from the psychological, the body from the mind.74 Indeed, she has taken up precisely Jonathan Lear’s challenge of understanding how vomiting could be thinking. Through exploring the melancholy and aggressiveness of various psychosomatic acts—such as regurgitation—she insists that the ‘other’ of the physiological within the physiological is recognised and put to work.75 Hers is a project which, unlike that of neuropsychoanalysts’ preoccupation with the embodied brain, pushes us to keep fantasy front and centre of any description of what the body is and does. Wilson, in her essay on neuropsychoanalysis, explicitly calls for the domains of biology, sexuality and fantasy to be conceptualised as ‘already native each to the other’, arguing that:

A psychosomatic structure built with already miscegenated components is an altogether different beast—conceptually, empirically, politically—from a psychosomatic structure that begins with discrete atomistic elements that only later come to be concatenated.76

In her investigation of how psychoanalysis and neuroscience might be thought together, she critiques many of the central formulations of neuropsychoanalysis for their overlooking of ‘lines of fissure’ between the neurosciences and psychoanalysis. She calls instead for a ‘mode of neuroscience–psychoanalysis that is strong because it is capable of tolerating, even enjoying and promoting, *the process of being unbuttoned*’ (italics added).77 Our article follows and expands on Wilson’s argument. We have previously argued that ‘current neuropsychoanalytic debates and exchanges make visible the *intractability* of moving between and across the axes of self-preservation and of Freudian sexuality’78—an intractability that we maintain should be embraced rather than covered over. While a number of 20th-century clinical investigations of the psychosomatic have indeed registered this intractability, neuropsychoanalysis has, Wilson laments, instead avoided ‘the wilder aspects of psychoanalytic theory’ and chosen ‘rectitude over variability’.79 This is, we think, one reason why clinical psychosomatic research—surely one of the ‘wilder aspects of psychoanalytic theory’—is rarely cited in neuropsychoanalytic texts. (This omission makes Solms’ own brief foray to the psychosomatic in his foreword to the volume by Stora all the more curious.)80

Neuropsychoanalysis is a scientific enterprise that claims for itself a space in which to understand motivated behaviours and, indeed, the embodied condition of being human. But despite its creativity and experimental vibrancy, neuropsychoanalysis, in its installation of a corrective biology as back story for the embodied brain, commits to an ideal, self-regulated subject ‘all the way down’—to use Monica Greco’s formulation.81 Neuropsychoanalysis too neatly cleaves dysregulation from regulation; too easily envisages motivation in terms of the priority of maintaining—in ways that are narrowly determined—life. The psychosomatic—that astonishing domain described in the 1890s by Freud and Breuer in their account of conversion hysteria—by contrast scrambles our usual understandings of the physiological, corporeal, psychological and mental. Greco, in her own contribution to this special issue, points to one of the most startling contributions of psychosomatic medicine: the proposition that an organism itself ‘is expressive of evaluations and sensitive to sociocultural values as part of its living milieu’.82 This demands a radical reconsideration of what adaptation might mean. Adaptation might well imply and involve organic disease or psychopathology, since addressing the psychosomatic demands addressing and considering a subject’s *own* aims and values in relation to the particularity of different environments. Our present moment—one in which we witness all manner of political, social and environmental emergencies—demands supple ways of imagining and practising forms of relationality that recognise how psyches, bodies and ecologies are sutured together as well as burst asunder.83 What drives someone into sickness of various kinds—and on occasion allows her to exit from such a state—requires capacious accounts of how we, as human subjects, suffer. So-called harmful—psychopathological—practices can be, in a fundamental sense, ways to adapt, and therefore ways to live.

Neuropsychoanalysis would no doubt imagine itself to be well on the way to answering Jonathan Lear’s question—‘How could vomiting itself be “thinking”’? But we might wonder what the price of entry has been, as the psyche is assimilated into the neuropsychoanalytic body.

## Notes

1. Jonathan Lear (1999), *Love and Its Place in Nature: A Philosophical Interpretation of Freudian Psychoanalysis* (New Haven, CT: Yale University Press), 7.

2. Sigmund Freud (1917), “Introductory Lectures on Psychoanalysis,” in *The Standard Edition of the Complete Psychological Works of Sigmund Freud*, trans. James Strachey, vol. XVI (London: Hogarth Press and The Institute of Psycho-Analysis), 258.

3. Felix Deutsch (1959), *On the Mysterious Leap from the Mind to the Body: A Workshop Study on the Theory of Conversion* (International Universities Press).

4. Jaak Panksepp and Mark Solms (2012), write that ‘[a]ffective states and subjective intentionality are intrinsic to the brain. They are part of nature, exerting causal effects’ (“What Is Neuropsychoanalysis? Clinically Relevant Studies of the Minded Brain,” *Trends in Cognitive Sciences* *Special Issue: Cognition in Neuropsychiatric Disorders* 16, no. 1 (1 January 2012): 6).

5. See Stefanos Geroulanos (2011), “The Brain in Abeyance: Freud and the Claim of Neuropsychoanalysis,”, *History of the Present* 1, no. 2: 219–43; Elizabeth A. Wilson (2011), “Another Neurological Scene,” *History of the Present* 1, no. 2: 149–69; Constantina Papoulias and Felicity Callard (2012), “The Rehabilitation of the Drive in Neuropsychoanalysis: From Sexuality to Self-Preservation,” in *Freuds Referenzen*, ed. Kirchhoff, Christina and Scharbert, Gerhard (Berlin: Kulturverlag Kadmos), 189–215; Nima Bassiri (2013), “Freud and the Matter of the Brain: On the Rearrangements of Neuropsychoanalysis,” *Critical Inquiry* 40, no. 1 (September 2013): 83–108; Elizabeth Rottenberg (2014), “Foreign Bodies: Psychoanalysis and Neuroscience,” *The Journal of Speculative Philosophy* 28, no. 3: 346–57.

6. Catherine Malabou (2012), *The New Wounded: From Neurosis to Brain Damage*, trans. Steven Miller (New York City, NY:Fordham University Press).

7. Siri Hustvedt (2011), *The Shaking Woman, or, A History of My Nerves* (London: Sceptre); see also Siri Hustvedt (2011), “Three Emotional Stories: Reflections on Memory, the Imagination, Narrative, and the Self,” *Neuropsychoanalysis* 13, no. 2(1 January 2011): 187–96.

8. Monica Greco, “On illness and value: biopolitics, psychosomatics, participating bodies,” *Med Humanit* 45, no. 2: 107–15

9. Sigmund Freud (1915), “Instincts and Their Vicissitudes,” in *The Standard Edition of the Complete Psychological Works of Sigmund Freud*, trans. James Strachey, vol. XIV (London: Hogarth Press and The Institute of Psycho-Analysis), 122.

10. Aikaterini Fotopoulou (2012), “The History and Progress of Neuropsychoanalysis,” in *From the Couch to the Lab: Trends in Psychodynamic Neuroscience*, ed. Aikaterini Fotopoulou, Donald W Pfaff, and Martin Conway (Oxford: Oxford University Press), 18.

11. For example, Morton Francis Reiser (1984), *Mind, Brain, Body: Toward a Convergence of Psychoanalysis and Neurobiology* (New York: Basic Books).

12. Sigmund Freud et al., 1990, *A Moment of Transition Two Neuroscientific Articles* (London and New York, NY: Karnac Books, 1990); Mark Solms (1997), *The Neuropsychology of Dreams: A Clinico-Anatomical Study* (Mahwah, NJ: L. Erlbaum Associates, 1997); Karen Kaplan-Solms and Mark Solms (2018), *Clinical Studies in Neuro-Psychoanalysis: Introduction to a Depth Neuropsychology*, second ed. (New York: Karnac, 2018); see the analysis in Geroulanos, ‘The Brain in Abeyance’.

13. See the published proceedings: International Neuro-Psychoanalysis Congress (2005), *Neuroscientific and Psychoanalytic Perspectives on Emotion*, ed. Maxine Jaffa (London: The International Neuro-Psychoanalysis Centre for the International Neuro-Psychoanalysis Society).

14. Mark Solms and Jaak Panksepp (2012), “‘The ‘Id’ Knows More than the ‘Ego’ Admits: Neuropsychoanalytic and Primal Consciousness Perspectives on the Interface Between Affective and Cognitive Neuroscience,” *Brain Sciences* 2, no. 2 (17 April 2012): 171.

15. Solms and Panksepp, 170.

16. E. R. Kandel (1999), “Biology and the Future of Psychoanalysis: A New Intellectual Framework for Psychiatry Revisited,” *American Journal of Psychiatry* 156, no. 4 (1 April 1999): 505–24. While Kandel did gesture to the body in that widely cited article—he writes of the ‘representation of body parts’ in the cerebral cortex, and research on the ‘development of the body plan’—the specific articulation of psyche and soma was not central to that article’s argument.

17. Edward Nersessian and Mark Solms (1999), “Editors' Introduction,” *Neuropsychoanalysis* 1, no. 1 : 3.

18. Edward O. Wilson (1998), *Consilience: The Unity of Knowledge* (New York: Knopf).

19. Nersessian and Solms, “Editors' Introduction,“ 3. This project of consilience has been critiqued byWilson, “Another Neurological Scene” and by Constantina Papoulias and Felicity Callard (2012), “The Rehabilitation of the Drive in Neuropsychoanalysis: From Sexuality to Self-Preservation.” More recently, the vision that is elaborated for neuropsychoanalysis has become less grandiose: Aikaterini Fotopoulou, for example, a central figure in the development of a ‘psychodynamic neuroscience’, warns that: ‘[g]iven that neither of the two facets [mental and neural] is sufficient to fully describe the actual phenomenon (the so-called “MindBrain” entity), collaboration and Dialogue [sic] may constrain and enhance each other’s models, without incorporating or eliminating each other’s unique scope and practice’ (see Aikaterini Fotopoulou (2012), “The History and Progress of Neuropsychoanalysis”, 23).

20. *International Neuro-Psychoanalysis Congress* *Neuroscientific and Psychoanalytic Perspectives on Emotion*, 23.

21. Notably, Solms said of Panksepp, at the special session of the congress of the International Neuropsychoanalysis Society devoted to Panksepp’s memory, that “I do not think [he] ever really understood a lot about psychoanalysis” (Bonny Astor et al. (2017), “Remembrances of Jaak Panksepp”, *Neuropsychoanalysis* 19, no. 2 (3 July 2017): 203). This is fascinating in terms of what it implies about the emergence of an interdiscipline: no substantial shared *knowledge* of the other epistemological domain was apparently required.

22. Solms and Panksepp, “The ‘Id‘ Knows More than the ‘Ego’ Admits: Neuropsychoanalytic and Primal Consciousness Perspectives on the Interface Between Affective and Cognitive Neuroscience,” 171.

23. Margaret R. Zellner et al. (2011), “Affective Neuroscientific and Neuropsychoanalytic Approaches to Two Intractable Psychiatric Problems: Why Depression Feels So Bad and What Addicts Really Want,” *Neuroscience and Biobehavioral Reviews* 35, no. 9 (October 2011): 2000–2008.

24. Jaak Panksepp (1998), *Affective Neuroscience: The Foundations of Human and Animal Emotions* (New York: Oxford University Press); see also Jaak Panksepp (2011), “Empathy and the Laws of Affect,” *Science* 334, no. 6061 (9 December 2011): 1358–59; Jaak Panksepp and Lucy Biven (2012), *The Archaeology of Mind: Neuroevolutionary Origins of Human Emotions* (New York: W. W. Norton).

25. Panksepp, “Empathy and the Laws of Affect,” 1358.

26. Panksepp capitalised these names to signal that they refer to a biological infrastructure of neural circuits rather than abstract concepts. We retain his convention here.

27. Jason S. Wright and Jaak Panksepp (2012), “An Evolutionary Framework to Understand Foraging, Wanting, and Desire: The Neuropsychology of the SEEKING System,” *Neuropsychoanalysis* 14, no. 1: 5–39.

28. Jaak Panksepp (2005), “Affective Consciousness: Core Emotional Feelings in Animals and Humans,” *Consciousness and Cognition, Neurobiology of Animal Consciousness*, 14, no. 1 (1 March 2005): 37.

29. Wright and Panksepp, “An Evolutionary Framework to Understand Foraging, Wanting, and Desire,” 21, 9.

30. Freud, “Instincts and Their Vicissitudes,” 121–22.

31. It is important to note here that the mapping of the drives onto Panksepp’s BESs is provisional, unstable and contentious for neuropsychoanalysts. However, certain articles authored by Solms and Panksepp (eg, Solms and Panksepp, “The ‘Id’ Knows More than the ‘Ego‘ Admits: Neuropsychoanalytic and Primal Consciousness Perspectives on the Interface Between Affective and Cognitive Neuroscience”; Mark Solms (2017), “What Is “the Unconscious,” and Where Is It Located in the Brain? A Neuropsychoanalytic Perspective,” *Annals of the New York Academy of Sciences* 1406, no. 1: 90–97), as well as the edited volume by Aikaterini Fotopoulou et al. (2012), *From the Couch to the Lab: Trends in Psychodynamic Neuroscience* (Oxford: Oxford University Press)), present a more settled account. This may be more to do with the function of such work in lending coherence to neuropsychoanalysis, rather than with a settlement of the question of the drive. However, it is precisely these kinds of texts that are more likely to circulate across disciplines, and we are therefore discussing them here. For a more detailed account of the neuropsychoanalytic difficulties accounting for the drives see Constantina Papoulias and Felicity Callard (2012) “The Rehabilitation of the Drive in Neuropsychoanalysis: From Sexuality to Self-Preservation”.

32. Sigmund Freud (1932), “New Introductory Lectures on Psychoanalysis,” in *The Standard Edition of the Complete Psychological Works of Sigmund Freud*, trans. James Strachey, vol. XXII (London: Hogarth Press and The Institute of Psycho-Analysis), 95.

33. Jean Laplanche and Pontalis J. B. (1968), “Fantasy and the Origins of Sexuality,” *International Journal of Psycho-Analysis* 49: 1–18.

34. Sigmund Freud (1905), “Three Essays on the Theory of Sexuality,” in *The Standard Edition of the Complete Psychological Works of Sigmund Freud*, trans. James Strachey, vol. VII (London: Hogarth Press and The Institute of Psycho-Analysis).

35. Sigmund Freud (1911), “Formulations on the Two Principles of Mental Functioning,” in *The Standard Edition of the Complete Psychological Works of Sigmund Freud*, vol. XII (London: Hogarth Press and The Institute of Psycho-Analysis), 215–226.

36. Sigmund Freud (1920), “Beyond the Pleasure Principle,” in *The Standard Edition of the Complete Psychological Works of Sigmund Freud*, vol. XVIII (London: Hogarth Press and The Institute of Psycho-Analysis).

37. Freud, “New Introductory Lectures on Psychoanalysis,” 105–106.

38. Two of the most powerful elaborations of this disjuncture between instinct and Freudian drive are Jean Laplanche (1985), *Life and Death in Psychoanalysis*, trans. Jeffrey Mehlman (Baltimore, MD: Johns Hopkins University Press); Leo Bersani (1986), *The Freudian Body: Psychoanalysis and Art*, Reprint edition (New York: Columbia University Press).

39. André Green (1999), *The Fabric of Affect in the Psychoanalytic Discourse*, trans. Alan Sheridan (London and New York: Routledge), 240.

40. Sigmund Freud (1938), “An Outline of Psycho-Analysis,” in *The Standard Edition of the Complete Psychological Works of Sigmund Freud*, vol. XXIII (London: Hogarth Press and The Institute of Psycho-Analysis), 148.

41. Mark Solms, “Extracts from the Revised Standard Edition of Freud’s Complete Psychological Works,” *The International Journal of Psychoanalysis* 99, no. 1 (2 January 2018): 32. Solms cites Panksepp here to substantiate this claim.

42. We have addressed this significant point in greater detail in a previous publication: see Constantina Papoulias and Felicity Callard (2012), “The Rehabilitation of the Drive in Neuropsychoanalysis: From Sexuality to Self-Preservation”.

43. It is worth noting here, that the other three BESs are not connected to the Freudian schema here. FEAR, RAGE and PANIC are, Solms tells us, *reactive* emotions and, as such, they are not activated by homeostatic imbalances, but by events in the world. Therefore, the regulation/satisfaction of a vital need is not relevant here.

44. Mark Solms and Jaak Panksepp (2012), “The “Id” Knows More than the “Ego” Admits: Neuropsychoanalytic and Primal Consciousness Perspectives on the Interface Between Affective and Cognitive Neuroscience”, 171.

45. While Solms explicitly acknowledges this at times, he generally sees this as a kind of cleaned-up Freud, or what we might call Freud 2.0.

46. Mark Solms and Margaret R Zellner (2012), “Freudian Affect Theory Today,” in *From the Couch to the Lab: Trends in Psychodynamic Neuroscience*, ed. Aikaterini Fotopoulou, Donald Pfaff, and Martin A Conway (Oxford: Oxford University Press), 136.

47. Solms and Zellner, 57.

48. See for example many of the contributions to Marilia Aisenstein (2010), *Psychosomatics Today: A Psychoanalytic Perspective*, 1st ed. (London: Routledge).

49. Margaret R. Zellner et al. (2011), “Affective Neuroscientific and Neuropsychoanalytic Approaches to Two Intractable Psychiatric Problems”.

50. Zellner et al. (2005, 2006).

51. Zellner et al. (2006).

52. Sigmund Freud (1898), “Sexuality in the Aetiology of the Neuroses,” in *The Standard Edition of the Complete Psychological Works of Sigmund Freud*, vol. III (London: Hogarth Press and The Institute of Psycho-Analysis), 259–85.

53. Zellner et al., “Affective Neuroscientific and Neuropsychoanalytic Approaches to Two Intractable Psychiatric Problems,” 2004.

54. Freud, “Sexuality in the Aetiology of the Neuroses,” 276. It is telling that this early Freudian text seized on here pre-dates the first iteration of the problematic of the drives and its attendant cleaving of sexuality away from the body’s biological needs.

55. Zellner et al., “Affective Neuroscientific and Neuropsychoanalytic Approaches to Two Intractable Psychiatric Problems,” 2006.

56. The fissure we identified earlier in the article that distinguishes psychoanalytic from psychological and behavioural accounts is thereby closed, even as neuropsychoanalytic accounts maintain that the objects of the BESs are, like the objects of the Freudian drive, not preset. In aiming towards what are called ‘real’ objects—that is, an engagement with the world that bears an adaptive signature—SEEKING is not about fantasy. The dimension of fantasy, which is indispensable to psychoanalysis, is elided in the distinction made between the real object and the objectless addiction.

57. Panksepp’s and Solms’ formulations on the BESs, and SEEKING in particular, emerge from a very specific reading of experiments on rats leading to the postulation and parsing of the elements of a reward system in the brain’s mesolimbic dopaminergic pathways. The constitutive role and repurposing of these experiments for neuropsychoanalysis and affective neuroscience alike merits further investigation.

58. Joseph Dodds, “Minding the Ecological Body: Neuropsychoanalysis and Ecopsychoanalysis,” *Frontiers in Psychology* 4 (25 March 2013): 1. See also: Brian Johnson and Daniela Flores Mosri (2016), “The Neuropsychoanalytic Approach: Using Neuroscience as the Basic Science of Psychoanalysis,” *Frontiers in Psychology* 7: 1459.

59. Solms, “Extracts from the Revised Standard Edition of Freud’s Complete Psychological Works”.

60. For example, see Mark Solms (1999), “Controversies in Freud Translation,”, *Psychoanalysis and History* 1, no. 1 (1 January 1999): 28–43.

61. Solms, “Extracts from the Revised Standard Edition of Freud’s Complete Psychological Works,” 17.

62. There are various theories of the ‘basic emotions’ beyond those of Panksepp; those elaborated by Paul Ekman have been particularly influential in the neurosciences. See Ruth Leys (2017), *The Ascent of Affect: Genealogy and Critique* (Chicago, IL: Chicago University Press). Mark Solms’s substitution of neuroscientific for psychoanalytic terms has occurred both when Freud’s own arguments are paraphrased, and in quotations from Freud himself. Solms and Nersessian argued, for example, that: ‘Freud believed that fixed patterns of affective motor discharge are, for the most part, innately prewired, although some basic emotions are apparently forged during early development by momentous biological events of universal significance’. When quoting from Freud’s ‘Inhibitions, Symptoms and Anxiety’, they substituted (in square brackets) ‘a basic emotion’ for Freud’s term ‘anxiety-state’ (Solms and Nersessian, “Freud’s Theory of Affect,” 9.) As readers of Freud will know, anxiety is one of the most labile and complex of affects in psychoanalysis, and one that perpetually crosses—translates—the mental to the somatic and back again. Its status as special kind of affect should on no account be mistaken for the ‘basic’ status of a scientifically demarcated ‘basic emotion’. To embed the term ‘basic emotion’ within Freud’s own metapsychological architecture is, then, a provocative step.

63. Jean Laplanche (1989), *New Foundations for Psychoanalysis*, trans. David Macey (Oxford: Basil Blackwell).

64. Mark Solms (2015), “Foreword,” in *A New Body-Mind Approach: Clinical Cases*, by Jean Benjamin Stora, trans. Sophie Leighton (London: Karnac Books), xiii.

65. Solms, ‘Foreword’. The phrase ‘functional system’ is the one that the psychologist Alexander Luria uses to describe the dynamic interactions of various neuronal mechanisms; it underpinned his shift to dynamic localization in the brain. Solms’ neuropsychological research is deeply indebted to the work of Luria.

66. Kaplan-Solms and Solms, *Clinical Studies in Neuro-Psychoanalysis: Introduction to a Depth Neuropsychology*.

67. Solms and Zellner, “Freudian Affect Theory Today,” 138.

68. Allan Young (2012), “The Social Brain and the Myth of Empathy,” *Science in Context* 25, no. 3 (September 2012): 401–24.

69. Jaak Panksepp and Lucy Biven (2012), “A Meditation on the Affective Neuroscientific View of Human and Animalian MindBrains,” in *From the Couch to the Lab: Trends in Psychodynamic Neuroscience*, ed. Aikaterini Fotopoulou, Donald Pfaff, and Martin Conway (Oxford: Oxford University Press), 152.

70. Panksepp and Biven, 160.

71. Panksepp and Biven, 161.

72. Solms and Zellner, “Freudian Affect Theory Today,” 138.

73. Wright and Panksepp, “An Evolutionary Framework to Understand Foraging, Wanting, and Desire,” 9, 5.

74. Elizabeth A. Wilson, *Psychosomatic: Feminism and the Neurological Body* (Durham NC: Duke University Press, 2004); Wilson, ”Another Neurological Scene”; Elizabeth A. Wilson (2015), *Gut Feminism* (Durham, NC: Duke University Press).

75. Wilson, *Gut Feminism*.

76. Wilson, “Another Neurological Scene,” 164.

77. Wilson, 150.

78. Papoulias and Callard, “The Rehabilitation of the Drive in Neuropsychoanalysis: From Sexuality to Self-Preservation,” 214.

79. Wilson, “Another Neurological Scene,” 156.

80. Solms, “Foreword.”

81. Greco, “On illness and value: biopolitics, psychosomatics, participating bodies,” 107–15.

82. Greco, 107–15

83. M. Y. Chen (2011), “Toxic Animacies, Inanimate Affections,” *GLQ: A Journal of Lesbian and Gay Studies* 17, no. 2–3 (1 June 2011): 265–86.
